# An Automated and Intelligent Medical Decision Support System for Brain MRI Scans Classification

**DOI:** 10.1371/journal.pone.0135875

**Published:** 2015-08-17

**Authors:** Muhammad Faisal Siddiqui, Ahmed Wasif Reza, Jeevan Kanesan

**Affiliations:** Faculty of Engineering, Department of Electrical Engineering, University of Malaya, Kuala Lumpur, Malaysia; CSIR-Institute of Microbial Technology, INDIA

## Abstract

A wide interest has been observed in the medical health care applications that interpret neuroimaging scans by machine learning systems. This research proposes an intelligent, automatic, accurate, and robust classification technique to classify the human brain magnetic resonance image (MRI) as normal or abnormal, to cater down the human error during identifying the diseases in brain MRIs. In this study, fast discrete wavelet transform (DWT), principal component analysis (PCA), and least squares support vector machine (LS-SVM) are used as basic components. Firstly, fast DWT is employed to extract the salient features of brain MRI, followed by PCA, which reduces the dimensions of the features. These reduced feature vectors also shrink the memory storage consumption by 99.5%. At last, an advanced classification technique based on LS-SVM is applied to brain MR image classification using reduced features. For improving the efficiency, LS-SVM is used with non-linear radial basis function (RBF) kernel. The proposed algorithm intelligently determines the optimized values of the hyper-parameters of the RBF kernel and also applied *k*-fold stratified cross validation to enhance the generalization of the system. The method was tested by 340 patients’ benchmark datasets of T1-weighted and T2-weighted scans. From the analysis of experimental results and performance comparisons, it is observed that the proposed medical decision support system outperformed all other modern classifiers and achieves 100% accuracy rate (specificity/sensitivity 100%/100%). Furthermore, in terms of computation time, the proposed technique is significantly faster than the recent well-known methods, and it improves the efficiency by 71%, 3%, and 4% on feature extraction stage, feature reduction stage, and classification stage, respectively. These results indicate that the proposed well-trained machine learning system has the potential to make accurate predictions about brain abnormalities from the individual subjects, therefore, it can be used as a significant tool in clinical practice.

## Introduction

In this modern era, the field of medical imaging proves its importance to increase in the need of automated and efficient diagnosis with some robustness. The use of computer based automatic systems in medical image processing, medical analysis, verification, and classification, is now widespread and highly helpful. In recent years, significantly advanced imaging tools (e.g., X-Rays, CT-Scans, and Magnetic Resonance (MR) imaging) are introduced in neurology and basic neuroscience fields, which enable in vivo monitoring of the brain. Magnetic Resonance Imaging is also known as MRI, has proven itself as a low risk, dominant, and flexible assessment technique for brain examination over the years because of its features, like better soft tissue differentiation, high contrast, and spatial resolution. MR image texture contains a rich source of information, which greatly increases the knowledge of the medical researcher to distinguish between the normal and diseased anatomy, and this information is also a very helpful component for any classification system [1–4]. MRI has emerged as one of the popular choices to rule out alternative causes of dementia and to detect a variety of brain conditions, such as tumor, bleeding, swelling, infections, cysts, inflammatory conditions, or problem with the blood vessels [5].

The radiologists’ conventional process for brain MR images classification is visual inspection. However, because of the huge amount of imaging data, the existing manual measurements of analysis and interpretation of these structures are tedious, time consuming, costly, subject to fatigue of the human observer, and do not capture the full pattern of atrophy. Hence, it generates the requirement of developing automated diagnostic systems for analysis and classification of such medical images. These intelligent systems can be a great instrument for the medical personnel in diagnosis, pre-surgical, and post-surgical procedures [6–10].

In the recently published work, various approaches of brain MRI classification have been proposed by different scholars. In general, most of the proposed systems consist of three sub-systems or phases. These phases are feature extraction, feature reduction, and classification. In [11], the authors have achieved 94% and 98% accuracy through classifiers based on self-organizing map (SOM) and support vector machine (SVM), respectively. They have used discrete wavelet transform (DWT) for feature extraction, but not used any feature reduction technique. The authors in [12], have used principal component analysis (PCA) with DWT for feature extraction and reduction. They have achieved 97% and 98% accuracy rates through feed-forward back-propagation neural networks (BPNN) and k-nearest neighbour (kNN) classifiers, respectively. Some other recent works by Zhang et al., [7, 13–15] have proposed different advanced methods for brain MRI classification and achieving high success rates. In all these schemes, they have used DWT and PCA for feature extraction and reduction, respectively. In [16], the authors introduced Ripplet transform (RT) for feature extraction with least squares support vector machine (LS-SVM) and achieved 100% accuracy for small datasets (having less number of samples as well as less number of diseases) and 99.39% accuracy for large datasets (having large number of samples and includes variety of diseases). However, the classification accuracies of most of the existing methods are greater than 90% for small datasets but it decreases for large datasets. Therefore, the goal is to achieve less complex and robust classifier system with high accuracy and will work on large datasets.

The main motivation of this work is to design high classification accuracy automated system with less computational complexity for brain MRI classification. The other incentive is to make the system more generalize so that it can work equally efficiently for different brain MRI datasets, consists of a varying number of disease classes. In this paper, fast DWT is used with PCA and LS-SVM. The fast DWT is used to compute only approximation feature of the images and PCA reduces the dimension of the features, which reduces the computation time. The categorization of images into normal or abnormal is done by the LS-SVM classifier, which automatically selects the appropriate parameter’s value of the kernel function by the proposed algorithm. Several recent literature accounted that higher classification accuracy can be achieved by the LS-SVM rather than the other existing data classification techniques [17, 18]. This is to separate the normal and abnormal images from brain MRI dataset with higher accuracy. Some of the other latest methods are also examined using the same datasets for comparative analysis.

The rest of the paper is organized as follows. Section 2 describes each block (feature extraction using fast DWT, feature reduction using PCA, and LS-SVM classification) of the proposed system. Details of the LS-SVM classifier are also explained in this section. Experimental results and comparisons are discussed in Section 3. Limitations of this study and future works are included in Section 4. Finally, conclusions are drawn in Section 5.

## Materials and Methods

**Fig 1:**
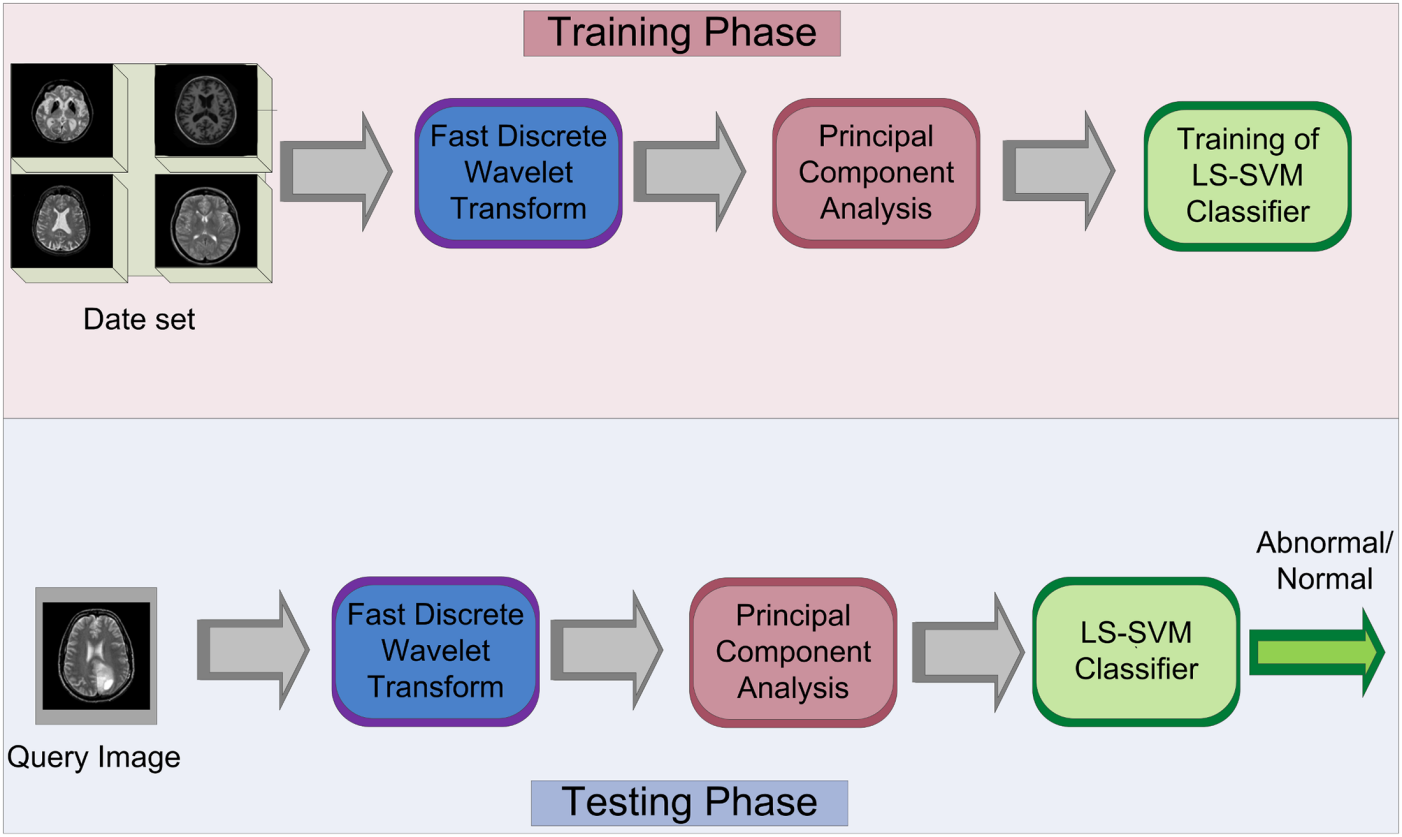
Proposed system methodology.

The proposed system is based on the following techniques: fast DWT, PCA, and LS-SVM. The main system is divided into two phases: training phase and testing phase. Fig 1 shows the block diagram and flow of the proposed design. MRI benchmark dataset is required initially to perform the classification, so the standard and generalized database is established by obtaining brain MR images from the ‘Harvard Medical School’ (http://med.harvard.edu/AANLIB/) and ‘Open Access Series of Imaging Studies (OASIS)’ (http://www.oasis-brains.org/). In the training and testing phases, first of all, fast DWT is computed which extracts the features of the images. Then, these images are processed by the PCA block for feature reduction. Finally, in the training section, LS-SVM classifier is trained by these reduced features. Meanwhile, the system is intelligent so that it gets the optimal values of the hyper-parameters of the radial basis function kernel for LS-SVM. Lastly, in the testing section, query image will be classified as a normal or abnormal.

### Feature Extraction Scheme

The prime objective of the feature extraction is to identify the relevant features in the image which leads to faster, easier, and better understand the images. The extracted features provide the characteristics of the input image pattern, which includes the major information about the images. The classifier will get only important features of the images after feature extraction, which highly improves its efficiency and accuracy, and reduces the computational time.

There are many algorithms used in previous research, such as DWT, RT, and some other techniques. DWT and its variant versions were used extensively by various scholars for feature extraction in brain MRI classification [7, 11–15, 19]. In [16], the authors used RT for feature extraction, which increases the complexity of the design and it is computationally expensive too. One of the characteristics of brain MRI is, it can be sparsified, so it can be represented in more sophisticated domains, such as wavelet domains [20]. Due to the sparse nature of brain image data, the wavelet transform represents rich information, thus, the DWT provides good feature extraction with less complex implementation and computation time.

#### 2-D fast Discrete Wavelet Transform

**Fig 2:**
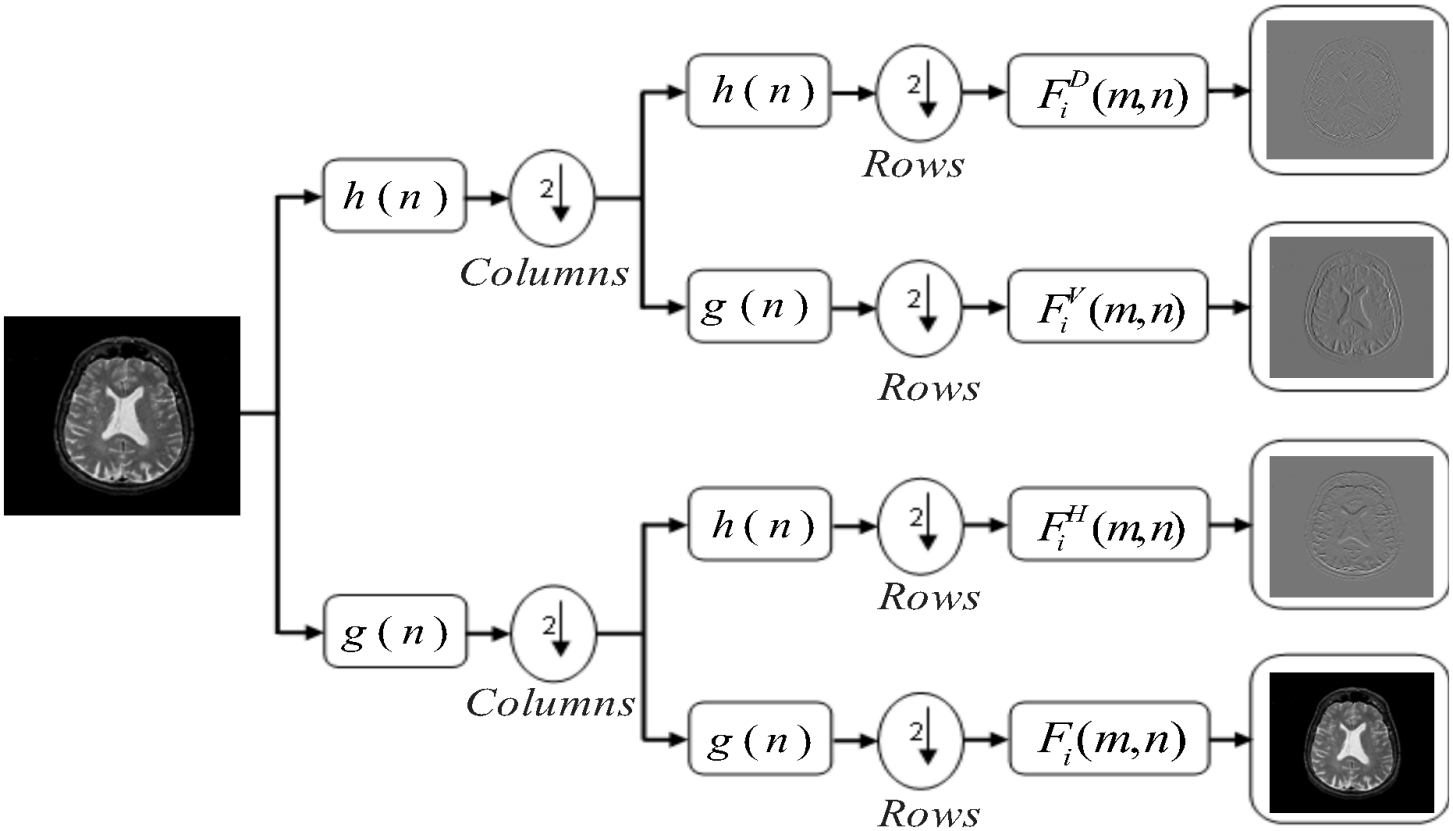
Schematic of 2D fast DWT.

The proposed work uses the 2-D fast DWT. It is a robust execution of the WT using the dyadic scales and positions [21]. The 2-D fast DWT is an iterative computational approach and can be expressed by Fig 2. The $F_{i}(m,n),\;F_{i}^{H}(m,n),\;F_{i}^{V}(m,n)$, and $F_{i}^{D}(m,n)$ are the approximation, horizontal, vertical, and diagonal coefficients in this figure, respectively. *F*_*i*_(*m*,*n*) gives the next approximation images computed by the fast DWT and *i* is the value of the decomposition level of wavelet transformation. Whereas, *m* and *n* represents the rows and columns in the image, respectively. The blocks containing the *g*(*n*) and *h*(*n*) are low-pass and high-pass decomposition filters, respectively. Finally, blocks containing ‘2’ with a down arrow illustrate down sampling by 2. The approximation component of the image can also be regarded as LL sub-band, while the horizontal (LH sub-band), vertical (HL sub-band), and diagonal (HH sub-band) can be regarded as the detailed components of the image. H represents a high pass filter operation on rows and columns of the image, while L corresponds to low pass filter applied on rows and columns of the image. For example, LL sub-band image is produced by: first columns are analyzed with low pass filter and then low pass filter is applied on rows. The approximation component will be treated as a mother component for the next iteration to find the next level of the decomposition. Mathematically, the equation for approximation (LL sub-band) can be derived from this iterative method of filtering and down sampling operations as:

$$S1_{i}(m,n)\;=\;F_{i\;-\;1}(m,n)*g(n)$$ (1)

$$S2_{i}(m,n)\;=\;{S1_{i}(m,n)|}_{n\;=\;2k\;;k\;\geq \;0}$$ (2)

$$S3_{i}(m,n)\;=\;S2_{i}(m,n)*g(n)$$ (3)

$$F_{i}(m,n)\;=\;{S3_{i}(m,n)|}_{m\;=\;2k\;;k\;\geq \;0}$$ (4)

Finally,

$$F_{i}(m,n)\;=\;{\left[\;g\;(n)*{\left\{\;F_{i-1}(m\;,n)*g\;(n)\;\right\}\;|}_{n\;=\;2k\;;k\;\geq \;0}\right]\;|}_{{}_{m\;=\;2k\;;k\;\geq \;0}}\text{ , }$$ (5)

where *i* = 1, 2, 3,…, *N*

In eq (5), *F*_*0*_(*m*,*n*) when *i* = 0 is the initial image and *F*_*i*_(*m*,*n*) gives the next approximation images computed by the fast DWT and *i* is the value of the decomposition level of wavelet transformation. In this paper, 3-level “Haar” wavelet transform is used to extract the features. Only LL sub-band is computed instead of implementing overall transformation, to make feature extraction faster in the proposed scheme compared to previous works. It significantly reduces the computational time of the proposed system without disturbing the accuracy of the classifier.

### Feature Reduction using Principal Component Analysis

Feature reduction is one of the basic components of any robust classifier, which reduces the massive database by measuring certain properties or features. Large database also increases the excessive features to classify that increases the computation time and storage memory. Furthermore, sometimes it increases the complexity of the classifiers, which is called curse of dimensionality. Therefore, it is necessitated to reduce the number of dimensions.

In feature extraction phase, the DWT also decreases the features, which shrink the large number of computational data. Brain MR image is represented by *F*(*m*,*n*) with the dimension of *m*-rows and *n*-columns. Normally, in brain MRI standard datasets, the images are squared, that means, *m = n*. After applying DWT for computing its approximation component, the size of the image will become: $\left(\frac{m}{2^{q}},\frac{m}{2^{q}}\right)$, here, *q* is the level of the wavelet transformation and $\frac{m}{2^{q}}\;<<\;m,$ where *q* = 3 in the proposed scheme. Therefore, the new image will be converted into $F_{a}\left(\frac{m}{2^{q}},\frac{m}{2^{q}}\right)$ and the dimension decreases. However, still the dimension is large enough; as a result, there is a need of more reduction in dimension.

One of the most popular and widely used techniques to reduce the dimension is PCA. PCA extracts the linear lower-dimensional representation of the data such that the variance of the reconstructed data is preserved [22, 23]. This technique has three characteristics: it provides that the elements of the input vectors are uncorrelated with each other; it sorts out the largest variation resulting orthogonal components in ascending order, and it discards the least variance components in the dataset. By using PCA, the dimension of the feature extracted image is more decreased from $\frac{m}{2^{q}}$ to the number of selected principal components. The number of selected principal components depends upon the ratio between the total variance of the original feature set and the total variance of the reduced feature set. Therefore, the main thought behind using PCA in our research is to further reduce the dimensionality of the fast DWT approximation feature components. This leads to provide efficient information to the classifier for making rapid and accurate decision.

### Support Vector Classification

SVM is an example of a supervised classification technique, offers an extremely efficient method of obtaining models for classifications [24, 25]. SVMs are supervised in the sense that they include a training session to learn the differences between two groups, which are going to be classified. This algorithm is structured on the theory of statistical learning, which helps in improving the general aptitude of machines to learn unseen data [26]. Recently, SVMs are widely used in many real-life applications, such as object detection [27], face identification in images [28], hand written alphabets recognition [29], and brain images abnormalities classification [15, 16, 30]. SVM classification is highly accurate and having elegant mathematical tractability than other classification techniques, like artificial neural networks, Bayesian networks, and decision tree. Latest research indicates that generally for the higher classification accuracy, an improved version of SVM, such as LS-SVM is remarkably better than the other existing algorithms [17]. In our proposed technique, LS-SVM classification is used due to its efficiency and robustness.

#### LS-SVM classification

The main drawback of SVM is overcome by the LS-SVM, which decreases the computational burden for huge-dimensional datasets. In LS-SVM approach, it manipulates the cost function of the typical SVM. This mathematical change simplifies the problem solution by introducing a least squares term in the cost function. Due to this improvement, the solution acquires from solving a set of linear equations, instead of the conventional method of solving quadratic programming for SVM [31, 32]. This approach significantly reduces the computation time and complexity of the classifier.

For the choice of kernel functions *K*(*x*_*k*_, *x*_*l*_) which satisfies the Mercer conditions and a new test sample point *x*, the LS-SVM classifier is given by (for mathematical details [31, 32]):

$$y(x)\;=\;sign\;\left[\sum_{i\;=\;1}^{N}\alpha_{i}\;y_{i}\;K(x,x_{i})\;+\;b\right]$$ (6)

Table 1 illustrates some of the common kernel functions used in LS-SVM. In our approach, LS-SVM is used with radial basis function (RBF) as a kernel function for training because previous literatures prove that RBF is a more compatible supported kernel function. RBF also reduces the computational complexity and improves the generalization performance of LS-SVM [17]. While using RBF as a kernel in LS-SVM, there are two tuneable hyper-parameters, which should be optimized accurately for achieving the best results. The hyper-parameters of RBF are *σ* and *γ*. The trade-off between margin maximization and error minimization is controlled by regularization parameter *γ*, while the kernel parameter *σ* determines the width of the kernel.

**Table 1:** Common kernel functions for LS-SVM.

Kernel Name	Function Expression
**Linear**	*K*(*x*, *y*) = *x* ^*T*^ *y*
**Polynomial**	$K(x,y)\;=\;{\left(1\;+\;\frac{x^{T}y}{\sigma^{2}}\right)}^{d}$
**RBF**	$K(x,y)\;=\text{ exp }\left\{-\;\frac{{\left\|x-y\right\|}^{2}}{\sigma^{2}}\right\}$
**SRBF**	$K(x,y)\;=\text{ exp }\left\{-\;\frac{\sum_{i\;=\;1}^{N}{\left(x\;-\;y\right)}^{2}}{\sigma^{2}}\right\}$

#### Hyper-parameters optimization and generalization of LS-SVM

For applying LS-SVM with any kernel function, one of the most important issues is to choose the hyper-parameters, which play a critical role in the performance of the classifier. With different parameters of the same kernel function, the LS-SVM prediction model has different performance. That means, the best optimization of hyper-parameters results the high accuracy of the classifier. Generally, for affecting learning and generalization of LS-SVM with RBF kernel, the two parameters, *σ* and *γ*, should be optimized.

There are many complex algorithms available for hyper-parameters optimization, namely, particle swarm optimization-based hyper-parameters selection [33], non-parametric noise estimator method [34], and grid search method [35]. Other than these excessively iterative methods, pilot run, is also used to find the values of these parameters by trial and error method. Mainly, for reducing the generalization error, the previous proposed schemes used different cross validation methods. The extreme case of cross validation has left-one-out (LOO), it provides almost an unbiased estimate of the generalization error but it is computationally very expensive. Meanwhile, the *k*-fold cross validation gives an excellent estimate of the generalization error at low cost [36]. In this paper, the less mathematically complex, comparatively less time consuming, and intelligent algorithm is used with *k*-fold cross validation. This algorithm, as shown below (Algorithm 1) gives the optimized value of the RBF-sigma (a more necessitated parameter for LS-SVM with RBF kernel) with *k*-fold cross validation, which makes more accurate, reliable, and generalize classifier.

**Algorithm 1.** Pseudocode of the RBF-sigma optimized value with *k*-fold cross validation method

Step 1: Initialization: *L*_min_, *ε*_min_, *σ*_*l*_, *σ*_*u*_, *k*, *i* = 1, *L*_*s*_ = 1 and *L* = 1

Step 2: Input one set of brain MR images from *k*-fold datasets *k*_*data*_(*i*)

Step 3: *σ*_*l*_ = *σ*_*l*_ + *L*

Step 4: Validation for certain parameter (*σ*_*l*_)

Step 5: Calculate *ε*

Step 6: if *ε < ε*_*min*_

  Obtain the certain parameter, *σ*_*k*_ = *σ*_*l*_

  if *i* < *k*

*k* = *k* + 1, *L* = 0 and go to Step 2

  else

   go to Step 7

  end if

 else

  if *σ*_*l*_ > *σ*_*u*_

   if *L* > *L*_min_

$L_{s}\;=\;\frac{L_{s}}{10}$, *i* = 1 and *L* = 1, update the value of *σ*_*l*_ and *σ*_*u*_ by lowest error interval and go to Step 3

   else

*i* = *i* + 1 and *L* = *L* + *L*_*s*_ go to Step 3

   end if

  end if

 end if

Step 7: $\sigma_{opt}\;=\;\frac{1}{k}\;\sum_{i\;=\;1}^{k}\sigma_{k}(i)$, obtain optimized and *k*-fold cross validated value of RBF-sigma

 In this algorithm:

*L*_*min*_ : Minimum tolerance in parameter value

*ε*_min_ : Tolerance, i.e., the minimum number of tests fails

*σ*_*l*_ : Lower bound of the parameter

*σ*_*u*_ : Upper bound of the parameter

*k* : Number of folds for cross validation

## Results and Discussion

The proposed technique is developed by using wavelet toolbox, image processing toolbox, and statistics toolbox of MATLAB software. The code can be tested or executed on any MATLAB compatible computer platform. The benchmark MRI database is evaluated by gathering the datasets from ‘OASIS’ and ‘Harvard Medical School’ MRI databases. The collected database consists of real human brain MR images. Both datasets consist of T1-weighted and T2-weighted MR brain images in the axial plane. The scan parameters used for these datasets are Voxel res: 1.0 × 1.0 × 1.25 (*mm*), Rect. FOV: 256/256, Orientation: Sag, TR: 9.7 (*ms*), TE: 4.0 (*ms*), TI: 20.0 (*ms*), and Flip Angle: 10°. The subjects are all right-handed and include both men and women scans. The dimensions of the images are 256 × 256 in a plane-resolution. The dataset consists of 340 patients’ brain MRI scans with the demographic and clinical details of the patient. These details include age, gender, clinical dementia rating (CDR), mini mental state examination (MMSE), and different test parameters.

**Fig 3:**
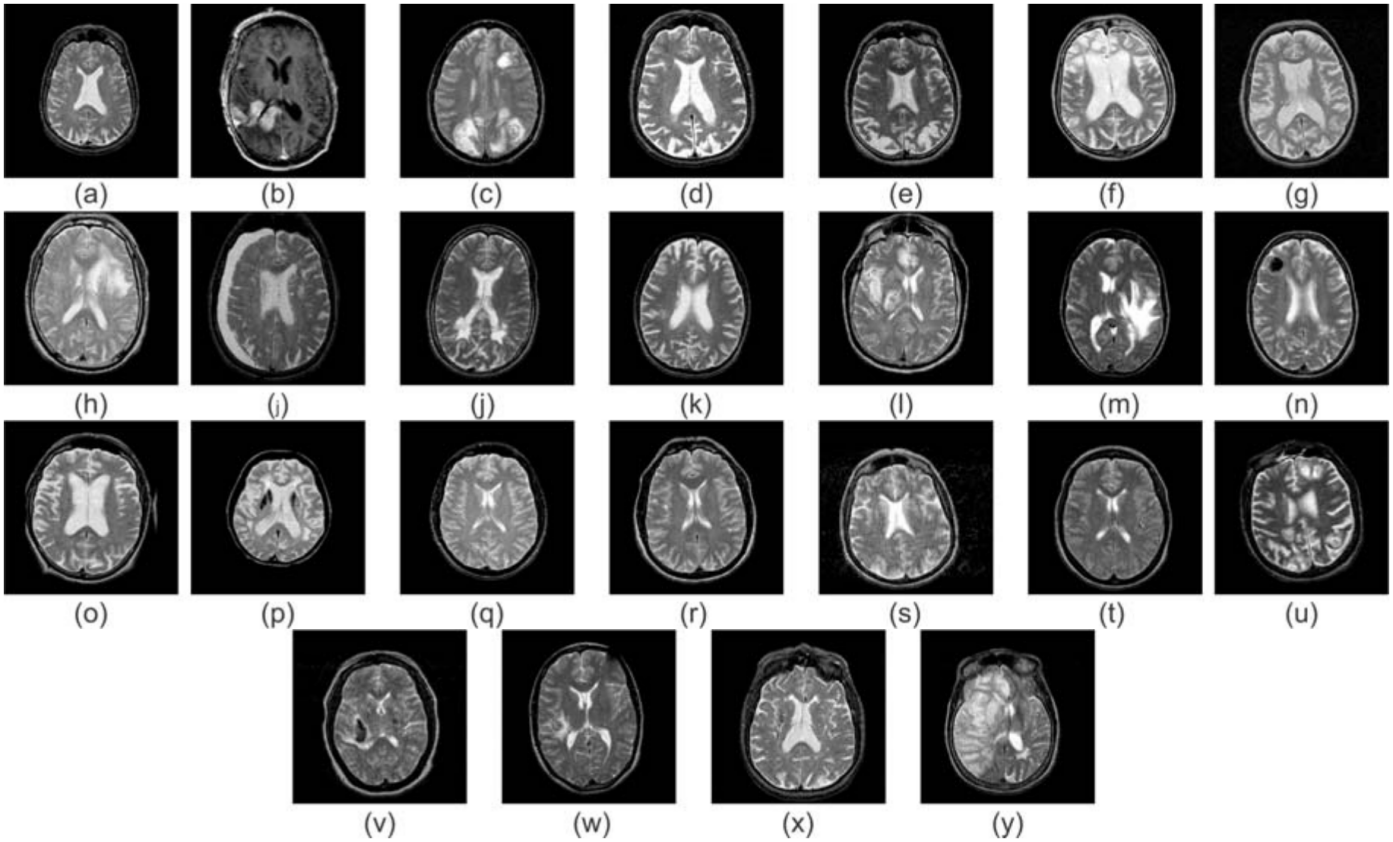
Sample images of various diseases in brain MRI dataset: (a) Normal brain (b) Glioma (c) Sarcoma (d) Alzheimer’s disease (e) Alzheimer’s disease with visual agnosia (f) Pick’s disease (g) Huntington’s disease (h) Meningioma (i) Chronic subdurnal hematoma (j) Multiple sclerosis (k) Cerebral toxoplasmosis (l) Herpes encephalitis (m) Metastatic bronchogenic carcinoma (n) Metastatic adenocarcinoma (o) Motor neuron disease (p) Cerebral calcinosis (q) AIDS dementia (r) Lyme encephalopathy (s) Creutzfeld-Jakob disease (t) Hypertensive encephalopathy (u) Multiple embolic infarctions (v) Cerebral haemorrhage (w) Cavernous angioma (x) Vascular dementia (y) fatal stroke.

The abnormal brain MR images are divided in two groups. First group (Group-1) has included 11 types of brain diseases, which are widely used as a benchmark dataset in previous studies. This group consists of normal brain images along with the following brain disease MRIs: glioma, sarcoma, Alzheimer’s disease, Alzheimer’s disease with visual agnosia, Pick’s disease, Huntington’s disease, meningioma, chronic subdurnal hematoma, multiple sclerosis, cerebral toxoplasmosis, and herpes encephalitis. The second more generalized benchmark dataset group (Group-2) having 24 types of diseases in total, among which, 11 types of diseases are the same as the previous group (Group-1) along with normal brain MRIs. The 13 new forms of abnormal images having the following diseases: metastatic bronchogenic carcinoma, metastatic adenocarcinoma, motor neuron disease, cerebral calcinosis, AIDS dementia, Lyme encephalopathy, Creutzfeld-Jakob disease, hypertensive encephalopathy, multiple embolic infarctions, cerebral haemorrhage, cavernous angioma, vascular dementia, and fatal stroke. The Group-2 dataset is more universal with 24 different diseases, which lead to test the classifier more comprehensively. The samples of each disease are shown in Fig 3.

The demographic information about the dataset is shown in Table 2. There is a total of 255 brain MR images in Group-1, which includes 220 abnormal and 35 normal MRIs. Group-2 is made up of 260 abnormal and 80 normal brain images (total of 340 brain MR images). Table 3 describes the settings of the training and validation images for the data groups. Validation images consist of a number of images from each subject of diseases and normal brain images. These images are not a part of the testing group images for unbiased validation of the classifier.

**Table 2:** Demographic information.

Group	Normal	Abnormal/Demented
**Age (mean, range) at MRI scan**	68.89 (33–94)	76.65 (62–96)
**Sex (F/M) (ratio)**	5/2	13/9
**MMSE score (mean, range)**	29.09 (25–30)	26.79 (14–30)

**Table 3:** Settings of training and validation images for dataset groups (one pass of 5-fold stratified cross validation).

Groups	Total no. of images		Total no. of training images		Total no. of validation images	
	Normal	Abnormal	Normal	Abnormal	Normal	Abnormal
**Group-1**	35	220	28	177	7	43
**Group-2**	80	260	27	100	53	160

The confusion matrix is widely used to determine the performance of the brain MRI classifiers. The sensitivity, specificity and accuracy of the proposed classifier is determined by the possible outcomes (*TP* (True Positive), *TN* (True Negative), *FP* (False Positive) and *FN* (False Negative)) [16] of the proposed decision suppot system.

In order to evaluate the performance of the proposed system in terms of feature reduction efficiency, sensitivity, specificity, accuracy, time analysis, comparisons with different high-tech schemes, and computation complexity, several experiments were performed on benchmark datasets of brain MRI. Before comparing the proposed method to other schemes, some methodological aspects are described here.

**Fig 4:**
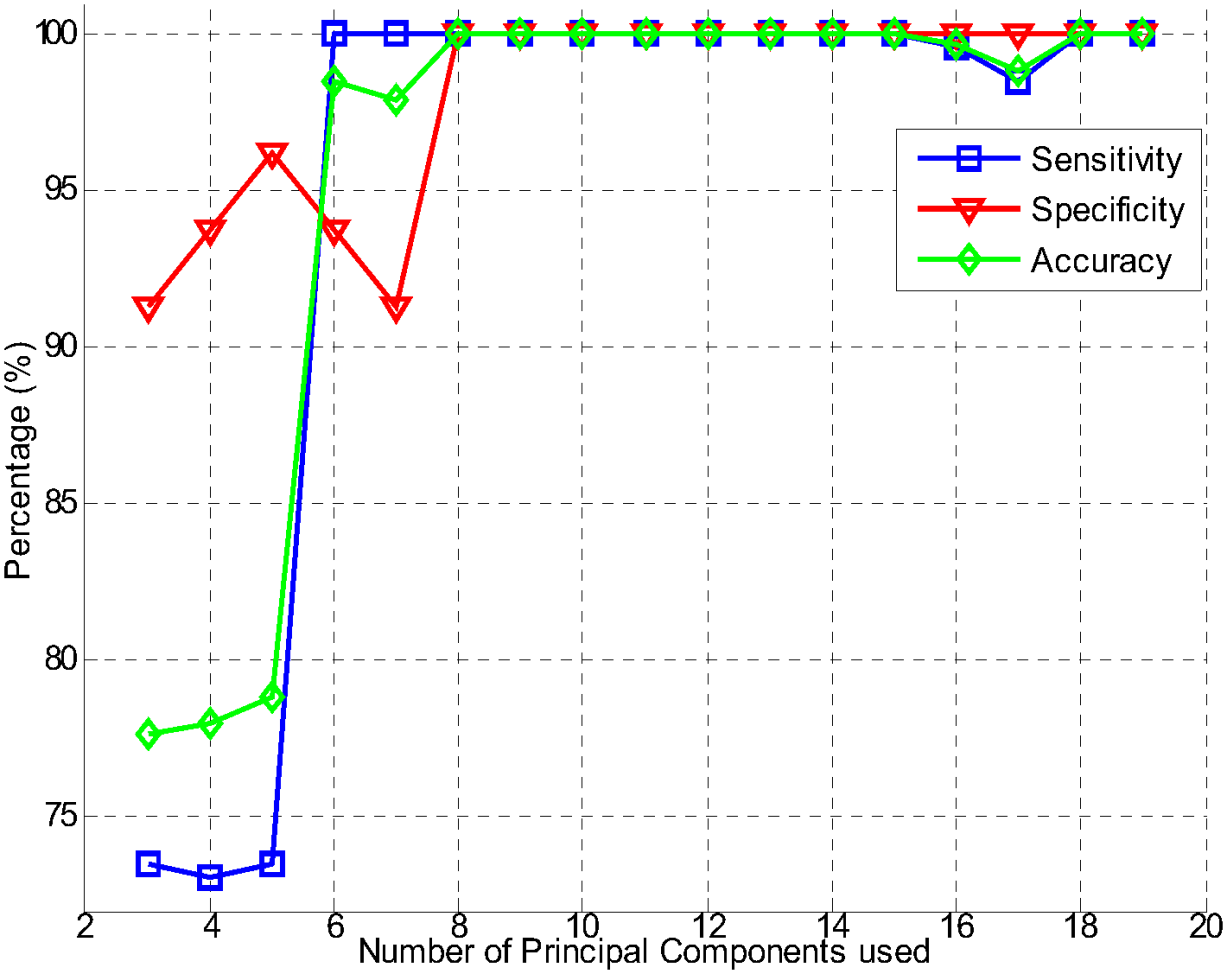
Sensitivity, specificity, and accuracy with respect to the number of principal components used.

**Fig 5:**
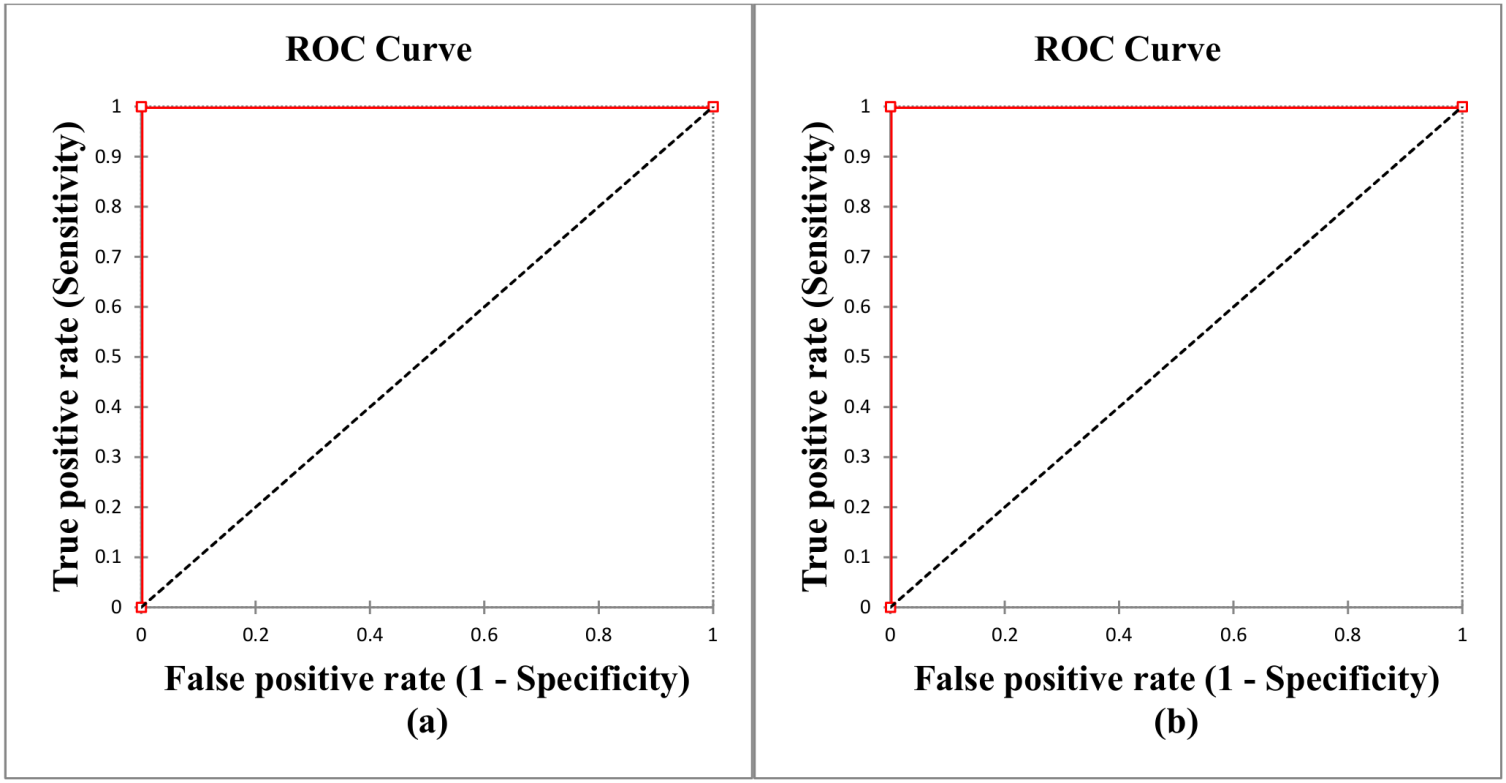
ROC curves of performance evaluation: (a) Group-1 and (b) Group-2.

This work uses fast DWT with some modifications, which reduces the computation time. The “Haar” wavelet transform is used for wavelet decomposition. The DWT decomposition configuration extracts the main features and also reduces the size of the brain MRI, which is initially 256 × 256 to 32 × 32. The PCA block uses these extracted feature vectors and gathers the high variance components. In this scheme, high success rate is achieved by using only 8 principal components. For classification purposes, the classifier is trained by only 0.012% and 0.78% of the original brain MRI and approximation components of the wavelet features, respectively. Therefore, due to this method, the system not only achieved 99.9% feature reduction, but also retains its high accuracy capability. This feature reduction achievement with a higher correctness rate is remarkably impressive than the other state-of-the art [16] brain MRI classification techniques. The performance of the proposed structure related to the number of principal components used is depicted by using different values of principal components in the experiments. Fig 4 shows the performance evaluation in terms of sensitivity, specificity, and accuracy, against the number of principal components used by the classifier. Number of the features may increase the complexity of the machine learning system to classify between two groups which eventually decreases the sensitivity and/or specificity of the system. It is easily found that our proposed system works extremely efficiently by using only 8 principal components for image presentation. The sensitivity, specificity, and accuracy are computed by observing the values of *TP*, *TN*, *FN*, and *FP* outcomes during the experiments. Fig 5 shows receiver operating characteristics (ROC) curves for evaluating the classification accuracy of the proposed system. The proposed system correctly classified the MR images of Group-1 and Group-2 with an average area under curve (AUC) of 100%, with 0% standard deviation.

For classification, we used LS-SVM with RBF kernel. Normally, many excessive computations and experiments are performed to get the best value of the hyper-parameters of the kernel. In recent published approaches, to estimate the suitable value of the parameters of the function, such as the order *d* in homogeneous polynomial (HPOLY) and inhomogeneous polynomial (IPOLY) kernel [15], the scaling factor *γ* in Gaussian radial basis (GRB) kernel [15], and the kernel and regularization parameters in LS-SVM [16], they use trial and error method iteratively by changing the value of the parameters manually. It takes hundreds of experiments to find the value of the kernel parameters. However, this method is really cumbersome, time consuming, and wasting the human resources. In this paper, a simple algorithm is therefore used to find the optimized value of the parameter that makes this system intelligent. It reduces the time as well as discovers the optimized value of the parameter. In our proposed work, the optimal value of the kernel parameter *σ* is determined by the proposed algorithm, i.e., 13.9, when keeping *γ* = 1. This optimized value is achieved at the cost of only a few experimentations. This value is not only optimized by our proposed algorithm, but also generalized by the use of *k*-fold cross validation in the algorithm. The algorithm used *k* = 5 for *k*-fold cross validation, to minimize the generalization error. Through all processes, we obtain the ranges of the kernel parameter *σ* ∈ [13.5, 15]and the regularization parameter *γ* ∈ [1, 3] on which maximum accuracy rate can be achieved. By using these optimized values of the parameters, we achieved 100% accuracy while testing different benchmark dataset groups and made our system a generalized one. The clinical efficiency of our classifier in terms of exactness is proven by the confusion matrix given in Table 4 (A: Actual, C: Classified).

**Table 4:** Confusion matrix of the proposed system.

	Group-1 Dataset		Group-2 Dataset	
	Normal (C)	Abnormal (C)	Normal (C)	Abnormal (C)
**Normal (A)**	28+7	0	27+100	0
**Abnormal (A)**	0	177+43	0	53+160

The performance of this work is compared with the latest 14 state-of-the-art brain MRI classification techniques, which are examined for the same MRI datasets (axial plane) and on the same platform. The proposed classifier is trained by axial plane brain images. However, different slices or plane (location) can also be classified by changing the training datasets accordingly. The comparison results are gathered after these experiments, as presented in Table 5. It indicates that the superior accuracy is obtained by our classifier system than the existing techniques. It can be observed that, the accuracy of RT + PCA + LS-SVM + RBF [16] is gradually decreasing when huge and versatile datasets are used, although it uses complex feature decomposition method. As reported in [11], DWT + SOM has the worst performance in terms of accuracy. The schemes proposed in [12], use the lowest feature dimension for classification, which is less than our proposed technique. However, Table 5 reveals that, the existing system [12], is less efficient than the proposed design in terms of correctness rate.

**Table 5:** Performance comparison using two different dataset groups.

Scheme	Feature Dimension	Accuracy (%)	
		Group-1	Group-2
DWT + SOM (Chaplot, et al., 2006)	4761	91.65	88.23
DWT + SVM + linear (LIN) (Chaplot, et al., 2006)	4761	94.05	90.29
DWT + SVM + POLY (Chaplot, et al., 2006)	4761	96.37	91.18
DWT + SVM + RBF (Chaplot, et al., 2006)	4761	96.18	90.88
DWT + PCA + forward neural network (FNN) (El-Dahshan, et al., 2010)	7	95.29	90.59
DWT + PCA + kNN (El-Dahshan, et al., 2010)	7	96.79	91.47
DWT + PCA + FNN + adaptive chaotic particle swarm optimization (ACPSO) (Zhang, et al., 2010)	19	97.38	94.41
DWT + PCA + FNN + scaled conjugate gradient (SCG) (Zhang, et al., 2011)	19	97.14	93.53
DWT + PCA + FNN + scaled chaotic artificial bee colony (SCABC) (Zhang, et al., 2011a)	19	97.81	94.71
DWT + PCA + kernel SVM (KSVM) + LIN (Zhang & Wu, 2012)	19	94.29	90.59
DWT + PCA + KSVM + HPOLY (Zhang & Wu, 2012)	19	95.61	91.47
DWT + PCA + KSVM + IPOLY (Zhang & Wu, 2012)	19	97.73	93.53
DWT + PCA + KSVM + GRB (Zhang & Wu, 2012)	19	98.82	94.11
RT + PCA + LS-SVM + RBF (Das, et al., 2013)	9	99.39	96.47
Fast DWT + PCA + LS-SVM + RBF (Proposed)	8	100	100

The feature dimension of [11], (4761 feature/image), is the worst case and also leads to the high computational complexity system. The methods described in [7, 13–15] use low features and show improved results in brain MRI classification. But, these methods are computationally complex because of using various complex weight optimization techniques. However, the proposed scheme requires only 8 feature vectors and obtaining the better accuracy among them. Moreover, by using only 8 dimensional feature vectors, the memory consumption is also reduced, which increases the efficiency of the classifier.

### Time Analysis Comparison

**Fig 6:**
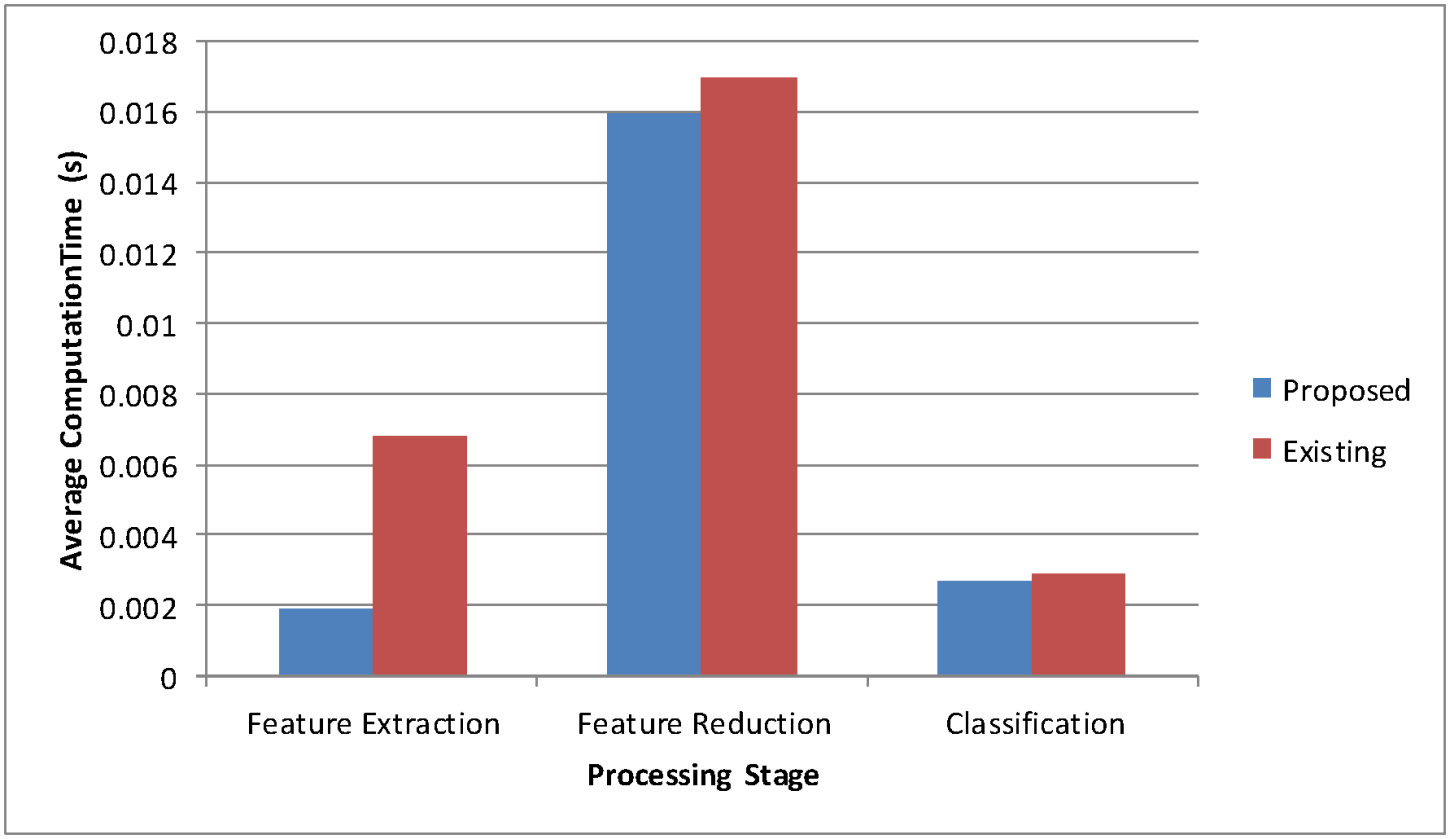
Time analysis comparison.

One of the other important performance measures is the computation time to evaluate the classifier. The time taken for the LS-SVM parameter optimization is not considered, although it is very low and training time is just 0.047s, since the parameters of the LS-SVM keep unchanged after training. All 340 images are tested through the proposed classifier and the computation time on all the stages (feature extraction, feature reduction, and classification) is recorded. For each brain MRI of 256 × 256 size, the proposed system consumes the average computation time in feature extraction, feature reduction, and LS-SVM classification of about 0.0019s, 0.016s, and 0.0027s, respectively. In comparison with the recent fastest version of the classifier [15], the existing system consumes 0.0068s, 0.017s, and 0.0029s for feature extraction, feature reduction, and SVM classification, respectively after executing on the same platform. Fig 6 shows the obtained results, which indicate that our proposed method improves the computation time by 71%, 3%, and 4% on feature extraction stage, feature reduction stage, and classification stage, respectively. The significant improvement in feature extraction stage is due to the modified fast DWT used for decomposition of the images. The total average computation time for testing of 256 × 256 size brain MR image is about 0.02076s (feature extraction time + feature reduction time + classification time), which has significant potential impact and it is demandable for real-world applications and for clinical decision support systems.

From the above discussed results and comparisons, it is clear that the proposed system has significantly high efficiency among all state-of-the-art literature works. Moreover, it is robust and it consumes lower computation time. It also demonstrates that the proposed system works equally efficiently with different sizes of datasets and various disease classes.

## Limitations and Future Works

This research proposes an intelligent, robust and accurate medical decision support system for classifying brain MRIs as normal or abnormal. The limitation of this system is that it is only validated for brain MRIs. However, the proposed intelligent system has the capability to classify any body parts MRI scans, once it is properly trained by appropriate datasets. In future, this work can be employed for different versions of MR images, such as proton density weighted and diffusion weighted images. Multi-classification aspect can be also explored, which would focus to classify the specific disease in brain MRI. The computation time could be decreased by applying the advanced wavelet transformation, such as the lift-up wavelet for feature extraction. LS-SVM with different novel kernels would be tested to increase the classification accuracy further. Other intelligent algorithms for kernel parameter optimization would be tested to improve the efficiency of the system. It can be also investigated for the effectiveness of other transforms along with supervised and un-supervised classification schemes for brain MRI classification. The extension of the developed scheme, to processing the other body parts’ MRI, is also a challenging issue of future research.

## Conclusion

In this study, the computer based medical decision support system is proposed for automatic classification of brain MR slices as normal or abnormal. This automated system is designed by the fast DWT, PCA, and LS-SVM method, which gives a promising accuracy in classifying the human brain as healthy or diseased. According to the experimental results, the proposed approach yielded better performance in terms of the minimum number of principal components used, sensitivity, specificity, classification accuracy, and time analysis, when compared to other popular methods available in recent literatures. The results stated that the proposed method is an accurate and robust classifier. The classification performance of this research work on different dataset groups with various diseases shows that it has an impressive generalization capability. It is evident from the time analysis that the proposed automated intelligent health care system easily meets the real time diagnosis challenges. Furthermore, it is easy to operate, non-invasive, efficient, and computationally inexpensive. The overall results indicated that, this automated classification tool can be easily equipped with medical imaging applications, which can assist the general practitioners to reach the final decision. Moreover, this intelligent system can be used for classification of images with different pathological conditions, types, and disease status.
